# Interactive Teaching Practices Among Trained and Untrained Medical Educators: A Cross-Sectional Analysis

**DOI:** 10.7759/cureus.97382

**Published:** 2025-11-20

**Authors:** Vimala Ananthy, Suresh Narayanan, Priyadharsini R Palanisamy

**Affiliations:** 1 Department of Pharmacology, Mahatma Gandhi Medical College and Research Institute, Puducherry, IND; 2 Department of Anatomy, All India Institute of Medical Sciences, Madurai, Ramanathapuram, IND; 3 Department of Pharmacology, Jawaharlal Institute of Postgraduate Medical Education and Research (JIPMER), Karaikal, IND

**Keywords:** active learning, faculty development programs, faculty training, interactive teaching, teaching preference

## Abstract

Background and aim: Competency-based medical curricula necessitate medical educators to incorporate interactive learning during lecture sessions. However, there is a lack of evidence on the impact of faculty training programs on educators' interactive teaching practices. Hence, this study compared the practices and beliefs of medical educators who had attended short-term and long-term educational training programs with those of educators who had not.

Material and methods: This questionnaire-based cross-sectional study was conducted among Phase 1 (pre-clinical) and Phase 2 (para-clinical) medical educators from multiple institutions. Data on educators' medical education training, perceived use of interactive teaching, preferred methods, difficulty levels of varied teaching strategies, perceived reasons for implementation, and challenges faced when implementing interactive teaching methods in lectures were collected. A total of 229 Phase 1 and Phase 2 medical teachers completed the survey, and their responses were analyzed.

Results: Educators who had participated in short-term and long-term medical education training programs have reported more frequent use of interactive teaching techniques compared to those who had not. Nonetheless, except for a few cases, the two groups were similar in their preferred interactive teaching methods, preferred methods for promoting higher-order thinking, difficulty levels of strategies, and perceived reasons for and barriers to implementation.

Conclusion: While educators who do not attend faculty development programs have similar perceptions of interactive teaching compared to those who attend training programs, engaging in training programs significantly increases the likelihood that an educator will utilize interactive teaching techniques in the classroom. This could be a multifactorial phenomenon or a reflection of the inherent nature of interactive teaching practice.

## Introduction

Faculty development programs (FDPs) refer to activities that enable educators to enhance their pedagogical knowledge and teaching skills [1]. Competency-based medical education emphasizes that faculties undergo training to augment their teaching skills [2]. In India, faculty development initiatives, such as Foundation for Advancement in Medical Education and Research (FAIMER), Advanced Course in Medical Education (ACME), Master's in Health Professional Education (MHPE), Postgraduate Diploma in Health Professional Education (PGDHPE), National Teachers Training Center (NTTC), Revised Basic Course Workshop (RBCW), and Curriculum Implementation Support Program (CISP), are used as platforms to improve medical teachers' teaching skills [3,4]. Of these programs, attending RBCW is mandatory for the promotion of medical faculties, whereas other programs are optional. Medical education unit (MEU) members must undergo ACME/PGDHPE/FAIMER/MHPE to act as facilitators of RBCW in their respective institutions. These training programs sensitize educators to active learning principles, acquaint them with varied interactive approaches, and demonstrate the integration of these strategies into their teaching process [3,5]. Despite such training programs, a few subsets of faculty resist active learning and prefer to teach in a didactic format, stating the challenges associated with interactive teaching.

In the Indian context, lectures remain the mainstay of delivering educational content, despite drawbacks such as audience passivity and diminished knowledge retention. Although several factors determine the implementation of active teaching in classrooms, FDPs play an important role [6]. In this context, researchers have attempted to analyze the effects of FDP using pre- and post-self-reported questionnaires or have reported the practices and beliefs of interactive teaching across institutions [7-14]. Studies examining the effects of FDP have observed that structured workshops and fellowship programs effectively improve educators’ teaching skills, confidence, and self-efficacy using self-reported satisfaction, attitudinal changes, and knowledge acquisition questionnaires [7,8]. Likewise, surveys among medical educators have reported on the proportion of them practicing active teaching, their preferred interactive teaching methods, and perceived reasons for and barriers to interactive teaching [9-11]. A few drawbacks of these studies include (1) the pre- and post-self-reported questionnaires focused on faculties' attitudinal change or knowledge acquisition immediately after the FDP and did not reveal whether those alterations were reflected in their classroom teaching, and (2) the self-reported indicators of FDP effectiveness were not compared with those of faculties who had not undergone any training programs. We often assume that medical educators who have undergone faculty training programs practice interactive teaching and have positive beliefs about it compared with those who have not. Hence, it is crucial to determine if the teaching practices and beliefs are linked to the FDP.

The self-efficacy theory deals with an individual's belief in their ability to perform specific tasks or challenges. This belief is influenced by (1) mastery experiences (past successes or failures), (2) vicarious experiences (modeling), (3) verbal persuasion (encouragement and feedback), and physiological and emotional states (stress, anxiety, and confidence) [15]. As training programs address all these elements, the self-efficacy theory was used as the conceptual framework in this study. This study hypothesized that there would be a significant difference in the proportion of faculties practicing interactive teaching and having perceived beliefs about it between those who have undergone fellowships/medical education training programs and those who have not. The primary objective of this study was to compare the following aspects between faculties who have undergone fellowship/medical education training programs and those who have not: (1) the proportion of faculties actively practicing interactive teaching, (2) the preferred choice of interactive teaching methods, (3) the teaching methods used to promote higher-order thinking, (4) the teaching methods perceived to be difficult, and (5) perceived reasons for and challenges in implementing interactive teaching. The secondary objective was to assess the impact of age, sex, and FDP training on the likelihood of practicing interactive teaching in lectures.

## Materials and methods

Study participants and setting

This questionnaire-based cross-sectional study was conducted over three months (October to December 2020). In India, basic sciences are taught in Phases 1 (Anatomy, Physiology, and Biochemistry) and Phase 2 (Pharmacology, Pathology, and Microbiology), whereas clinical subjects are taught in Phases 3 (Otorhinolaryngology, Ophthalmology, and Community medicine) and Phase 4 (Medicine, Surgery, Obstetrics and Gynecology, and Paediatrics) of the medical curricula. The study population included MBBS Phase 1 and Phase 2 educators affiliated with medical colleges across India (purposive sampling). The teaching practices of Phase 1 and Phase 2 medical educators were specifically evaluated because the basic science curriculum relies heavily on didactic lectures compared with Phase 3 and Phase 4, where most of the course content is delivered via clinical posting. The study participants were educators with varied teaching experience, such as professors, associate professors, assistant professors, senior residents, and tutors. During the literature review, details regarding the proportion of faculties practicing interactive teaching based on their FDP training could not be located. Hence, a pilot study (n = 50; 25 without FDP, 25 with FDP) was done and yielded the following results: 17/25 (68%) of faculty without FDP and 22/25 (87%) of faculty with FDP routinely practiced interactive teaching. Subsequently, using the Statulator online software, a sample size of 83 per group was derived, assuming a 95% confidence level and 80% power.

Ethical considerations

Ethical committee approval was obtained before commencing the study (IRC/04/2020/71/IHEC/162). The Google Form (Google Inc., Mountain View, CA) used for the survey was structured, with the first section containing the study objective and a consent form. Only if the participants consented would the survey questionnaire pop up. The participants' anonymity and data confidentiality were ensured throughout the data collection process. 

Data collection tool

A comprehensive literature review was performed by two senior medical education unit members and three faculties from Anatomy, Pharmacology, and Microbiology to identify the commonly used interactive teaching methods and questions to determine the practices and beliefs of medical educators in the Indian context. We considered interaction as any teaching method that promotes active discussion among peers or between students and the learning environment. In the collected data, the four/five most common responses were selected as options for the questions to simplify the statistical analysis. Then, five medical education experts vetted the questionnaire for construct and content validity. The panellists rated the questions on a five-point scale (5 -- very high significance; 1 -- very low significance). Items showing ≥75% agreement and a median score of 4 or 5 were added, whereas those scoring <75% agreement were excluded. The questionnaire was given to seven medical educators to check for reliability (Cronbach's alpha = 0.8).

The survey instrument comprised two sections. The first section focused on participants' demographic details (gender and designation) and information on their medical education fellowship or workshop experience. Short-term (three to seven days) and longitudinal educational training (one to two years) programs were included as these inculcate information about interactive teaching methods, the advantages/disadvantages of these techniques, and hands-on experience with the teaching methods.

Leadership/research/statistics/simulation training programs, educational webinars, and lectures spanning one to three hours were excluded because these programs might not directly influence the interactive teaching practice. The second section involved six semi-open questions with multiple-choice options. For item 1, the options to determine the proportion of faculties practicing interactive teaching were “never, rarely, occasionally, frequently, and always.” Rather than asking questions in the “yes” or “no” format, a Likert-scale assessment was employed, as it would genuinely reflect the teaching practice. As our research question was to compare the proportion of faculties practicing interactive teaching, “never,” “rarely,” and “occasionally” responses were considered as not practicing interactive teaching, and “frequently” and “always” as practicing it. For items 2-4, the names of the interactive teaching methods (oral questions, quiz, think-pair-share, handouts, concept maps, and clickers) were given as options to determine the preferred method of interactive teaching, the preferred technique to promote higher-order thinking, and the teaching method perceived to be challenging to implement. For items 5 and 6, the perceived reasons for implementation (break the monotony, motivate students to engage in class discussions, encourage critical thinking, and ensure better knowledge retention) and barriers/difficulties faced (time constraints, large group, lack of technical support, requires extra preparation, lack of knowledge, and lack of motivation) while conducting the interactive sessions were provided as options [10]. The option “others” was provided for all questions, allowing participants to supplement their responses with their perspectives. Google Forms were shared in 10 medical education and seven subject-specific WhatsApp groups. A reminder was sent after three weeks, and Google Forms were closed after four weeks as an adequate sample size was reached. The data were downloaded into a Microsoft Excel sheet (Microsoft Corporation, Redmond, WA), and duplicate entries and partly filled data were removed. Participants' responses were coded and analyzed. 

Data analysis

The data were analyzed using the Statistical Package for the Social Sciences (SPSS) software, Version 23 (IBM Corp., Armonk, NY). Data on the practices and beliefs of medical educators on interactive teaching were expressed in proportions. The chi-square test was used to estimate the differences in interactive teaching practices, choice of interactive teaching methods, and perceived reasons for and challenges in implementing interactive teaching between FDP-attended and not-attended educators. A p-value of <0.05 was considered statistically significant. Binomial regression analysis was employed to explore the variables influencing the frequency of interactive teaching usage. Age, sex, and attending FDP were the variables considered in the regression analysis, and the outcome variable included the likelihood of practicing interactive teaching.

## Results

A total of 229 responses were collected via the survey. Of the participants, 88 (38.43%) had not attended training programs, whereas 141 (61.57%) had participated in such programs. The demographic details, categorized by sex and designation, are furnished in Table 1. The educators who had attended training programs reported that they more frequently used interactive teaching techniques compared to those who had not (Table 2). However, except for a few cases, significant differences were not observed in the preferred choice of interactive teaching method, the preferred choice of the teaching method in promoting higher-order thinking, the difficulty level of the strategies, and reasons for and barriers to implementation between the FDP-attended and not-attended groups (Table 2). Two parameters, quizzing strategy use and perceived lack of knowledge, exhibited significant differences between the two groups (Table 2). The logistic regression model was statistically significant, χ^2 ^(3) = 15.775, p < 0.05. The model explained 9.3% (Nagelkerke R2) of the variance in the likelihood of practicing interactive teaching in lectures and correctly classified 67.2% of the cases. The odds ratio of age, sex, and FDP training on the possibility of practicing interactive teaching in lectures was derived (Table 3).

**Table 1:** Demographic details of the study participants. FDP: faculty development programs.

		FDP not attended faculties (N = 88)	FDP attended faculties (N = 141)
Gender	Male	30 (34.1%)	61 (43.2%)
	Female	58 (65.9%)	80 (56.8%)
Designation	Professor	3 (3.41%)	37 (26.24%)
	Associate professor	9 (10.23%)	30 (21.28%)
	Assistant professor	58 (65.91%)	65 (46.10%)
	Senior resident	13 (14.77%)	8 (5.67%)
	Tutor	5 (5.68%)	1 (0.71%)

**Table 2:** Comparison of teaching preferences and beliefs among FDP-attended and not-attended faculties. *p-value of <0.05 indicates a significant difference. FDP: faculty development programs.

Parameters	Faculties who have not attended educational training programs (N = 88)	Faculties who have attended educational training programs (N = 141)	Chi-square statistic (χ²)	p-value
1. Frequency of interactive teaching use (never, rarely and occasionally – not using interactive teaching, frequently and always – using interactive teaching)				
Interactive teaching practice	48 (54.5%)	108 (76.6%)	12.13	<0.01*
2. Preferred choice of interactive teaching methods				
Oral questions	72 (81.8%)	126 (89.4%)	2.63	0.10
Quiz	25 (28.4%)	59 (41.8%)	4.21	0.04*
Think-pair-share	14 (15.9%)	35 (24.8%)	2.56	0.11
Handout	18 (20.5%)	31 (22%)	0.76	0.78
Concept maps	14 (15.9%)	28 (19.9%)	0.56	0.45
Clickers	5 (5.7%)	15 (10.6%)	1.67	0.19
3. Interactive teaching method used to promote higher-order thinking				
Think-pair-share	47 (53.4%)	81 (57.4%)	0.35	0.55
Oral questions	30 (34.1%)	47 (33.3%)	0.01	0.91
Quiz	23 (26.1%)	25 (17.7%)	2.31	0.13
Concept maps	12 (13.6%)	19 (13.5%)	0.001	0.97
Handout	7 (8%)	12 (8.5%)	0.02	0.88
Clickers	9 (10.2%)	8 (5.7%)	1.63	0.20
4. Interactive teaching method perceived to be difficult to implement				
Think-pair-share	34 (38.6%)	45 (31.9%)	1.08	0.29
Clickers	21 (23.9%)	30 (21.3%)	0.20	0.65
Quiz	20 (22.7%)	28 (19.9%)	2.69	0.60
Handout	17 (19.3%)	18 (12.8%)	1.79	0.18
Concept maps	5 (5.7%)	10 (7.1%)	0.17	0.67
Oral questions	7 (8%)	4 (2.8%)	3.10	0.08
5. Perceived reasons for implementing interactive teaching				
Break monotony	61 (69.3%)	108 (76.6%)	1.48	0.22
Motivate students in class discussions	47 (53.4%)	91 (64.5%)	2.80	0.09
Encourage critical thinking	45 (51.1%)	90 (63.8%)	3.60	0.06
Better knowledge retention	46 (52.3%)	84 (59.6%)	1.17	0.28
6. Perceived challenges while implementing interactive teaching				
Time constraints	59 (67%)	96 (68.1%)	0.02	0.87
Large group	47 (53.4%)	91 (64.5%)	2.80	0.09
Lack of technical support	32 (36.4%)	60 (42.6%)	0.86	0.35
Requires extra preparation	25 (28.4%)	30 (21.3%)	1.51	0.21
Lack of knowledge	26 (29.5%)	16 (11.3%)	11.98	0.001*
Lack of motivation	12 (13.6%)	15 (10.6%)	0.46	0.49

**Table 3:** Logistic regression analysis of age, gender, and attending FDP over the practice of interactive teaching. *p-value of <0.05 indicates a significant difference. FDP: faculty development programs.

Variables	B	Odds ratio Exp(B)	95% Confidence interval	P-value
Age	0.04	1.04	0.99-1.09	0.064
Gender	0.07	1.08	0.59-1.95	0.795
Attended FDP	0.80	2.232	1.21-4.11	0.010*

## Discussion

This study identified that Phase 1 and Phase 2 educators who had participated in short-term and long-term training programs reported that they more frequently utilized interactive teaching techniques. Nonetheless, the two groups did not differ significantly in terms of their preferred interactive teaching methods, preferred methods for promoting higher-order thinking, the difficulty level of the strategies, and reasons for and barriers to implementation, except for a few cases. 

Burdick et al. reported that longitudinal fellowship courses, such as FAIMER, enhanced faculty knowledge/skills in teaching methodology, which could be applied at their institutions [16]. Similarly, Singh et al. analyzed longitudinal FDP using the quasi-experimental methodology. The researchers documented an increase in the self-efficacy beliefs of faculties, such as communication, classroom management, motivation of students, accommodation of individual differences, and higher-order thinking skills [17]. The current investigation differs from previous studies in the following aspects: (1) a valid questionnaire reflecting the actual classroom practice was used, (2) faculties who had not attended FDP were used as a control group, and (3) survey responses were solicited from instructors who had undertaken longitudinal fellowships as well as from those who attended short-duration medical education workshops. FDP-attended faculties were observed to be 2.23 times more likely to practice interactive teaching than not-attended faculties. In medical education training programs, the participants discuss and get trained on interactive teaching methods, align the interactive teaching method with a given objective, design educational projects focused on interactive teaching methods, and share their perspectives in forum deliberations [3,5]. During this process, educators might develop positive attitudes toward interactive teaching or gain hands-on knowledge [8,18]. Similar findings have been recorded by Desselle et al., where a one-hour faculty development program enhanced faculties' utilization of active learning strategies during lectures, as measured via questionnaires and direct observation of lectures [19]. Although previous studies have stated that age and sex influence the practice of active teaching [9], such an association was not observed in the regression analysis performed in this study.

Rahman noted that FDP participants were more efficacious in course design, classroom management, instructional strategy, assessment, and technology adoption than non-participants [18]. In this study, interactive teaching methods practiced by the participants were specifically collected to obtain an in-depth understanding of classroom interaction. Significant differences were evident in two parameters: the higher use of the quizzing strategy among the FDP-attended group and the perceived lack of knowledge among the non-attended group. Vyas et al. noticed flaws in posing multiple-choice questions (MCQs) primarily because of a lack of faculty training [20]. In medical fellowship courses, faculties are divided into small groups to discuss the advantages of MCQs over other assessment modalities, guidelines to frame MCQs, and the importance of formative assessment [16,17]. Similarly, in RBCW, two to three hours are dedicated to sensitizing the faculties on the various components of MCQs, framing a single best type of MCQ, and the need to evaluate the student's knowledge [21]. The perceived higher use of the quizzing strategy among the FDP-attended group could be attributed to the increased confidence in framing the quiz or the enhanced motivation to test the student's acquired knowledge [22]. The lack of significant differences between the two groups in other teaching modalities indicates the ubiquitous nature of the teaching strategy, which does not need specific training. Medical educators undergoing longitudinal fellowships must complete a medical education project as part of the fellowship. Hence, they are exposed to diverse interactive teaching formats while researching for their project. Furthermore, the educators gather input from their colleagues during project discussions and their senior fellows during project presentation sessions [3,16,17]. As faculties who had not attended FDPs lack such opportunities, it is unsurprising that a significantly greater number of them reported a lack of knowledge. This finding indicates that not-attended educators are either unaware of the interactive teaching trends or do not possess the methodological expertise to conduct interactive sessions.

A systematic review of FDPs reported positive attitudes toward teaching and self-reported changes in teaching behaviors but limited alterations in organizational practice [8]. In contrast, another study observed that medical education fellowship programs positively impacted organizational development [23]. In our study, FDP participants who attended and those who did not differ significantly in their preferred interactive teaching methods, preferred methods for promoting higher-order thinking, the difficulty level of the strategies, and reasons for and barriers to implementation, except for a few cases. The following are the possible explanations for this finding: 1) The educators who had not attended FDPs might have acquired pedagogical knowledge via self-interest, peer shadowing, and institutional guidelines [24,25]. FDP not-attended teachers might have obtained their pedagogical skills via one of the potential factors, and exactly how educators gained the practice of interactive teaching is beyond the scope of the study. 2) Investigations have suggested that the change in educators’ behavior after attending the FDP depends upon adequate support provided by the program, the willingness of the educator to change their teaching practice, and a fostering workplace [26,27]. 3) In addition, the preferences and beliefs could reflect the inherent nature of interactive teaching. For instance, FDP-attended and not-attended faculties have reported questioning as their preferred mode of interaction. This approach demands less pre-lecture preparation and is easy to implement [28]. The inherent nature of the teaching method, rather than FDP training, might be an essential factor in the perceived preference. Moreover, participants in both groups felt that time constraint was a factor that hindered interactive teaching. This finding is expected as interactive teaching requires class time to provide a trigger, synthesize student opinions, share their opinions in the classroom, and engage in faculty discussions [29]. The training programs could play a minimal role in reducing the time required to implement interactive teaching.

Strengths and limitations

The strength of this study is the inclusion of participants from multiple institutions with diverse teaching experiences. Compared with previous studies focusing on the extent and perceptions of active learning use among educators [10,11], this study analyzed the practices and beliefs of interactive teaching among FDP-attended and non-attended educators. However, this study has certain limitations, which include (1) the self-reporting nature of the survey rather than the actual observation of educators' teaching practice, (2) response bias: some faculties might not be familiar with the exact terminologies of interactive teaching methods, and (3) the inability of the study design to determine whether the faculties' attainment of professional competence is exclusively due to FDP or gained over the course of their teaching experience. Furthermore, our study population represented only a subset of medical educators, which impairs our ability to generalize the results across healthcare professionals.

Implications of the study

This study has documented the hierarchical preferences and beliefs of interactive teaching methods in lectures among FDP-attended and non-attended educators. Researchers can utilize the findings as a reference to monitor the changes in organizational teaching practice and educators’ beliefs about interactive teaching. The results suggest that structured training programs change educators' teaching practices by encouraging them to implement interactive teaching techniques more frequently, emphasizing the need for continuous professional development for educators [7,8,19]. In addition, this research has established that educators' preferences and beliefs regarding interactive teaching could be a multifactorial phenomenon and might not depend exclusively on FDP training.

## Conclusions

This study has demonstrated that educators who engage in faculty training programs are more likely to use interactive teaching techniques in lectures. Nonetheless, educators' choice of teaching methods, perceived reasons for implementation, and challenges in interactive teaching remain the same regardless of the training programs. 

## Appendices

Section A: Demographic information

Gender*

☐ Female

☐ Male

Age*

(Numerical value)

Designation*

☐ Tutor/Demonstrator

☐ Assistant Professor

☐ Associate Professor

☐ Professor

☐ Other: (Specify)

Have you attended any training program in teaching-learning methods in medical education? *

☐ Revised Basic Course in Medical Education (RBC)

☐ Advanced Course in Medical Education (ACME)

☐ National Teacher Training Centre (NTTC) Course

☐ Postgraduate diploma in health professional education

☐ Masters in health professional education

☐ FAIMER

☐ None

☐ Other: (Specify)

Section B: Practice and perception of interactive teaching

How often do you use interactive teaching methods in your lectures? *

☐ Always

☐ Frequently

☐ Occasionally

☐ Very rarely

☐ Never

Preferred interactive teaching methods (You may select more than one)

☐ Oral questions

☐ Quiz

☐ Think-Pair-Share

☐ Handouts

☐ Concept maps

☐ Clickers

☐ Others

Preferred teaching methods used to promote higher-order thinking (You may select more than one)

☐ Think-Pair-Share

☐ Oral questions

☐ Quiz

☐ Concept maps

☐ Handouts

☐ Clickers

☐ Others

Interactive teaching methods perceived to be difficult to implement (You may select more than one)

☐ Think-Pair-Share

☐ Clickers

☐ Quiz

☐ Handouts

☐ Concept maps

☐ Oral questions

☐ Others

Perceived reasons for implementing interactive teaching (You may select more than one)

☐ Break monotony

☐ Motivate students to participate in class discussions

☐ Encourage critical thinking

☐ Enhance knowledge retention

☐ Others

Perceived challenges while implementing interactive teaching (You may select more than one)

☐ Time constraints

☐ Large group size

☐ Lack of technical support

☐ Requires extra preparation time

☐ Lack of knowledge of methods

☐ Lack of motivation

☐ Others
